# *Trans*-packaging of human immunodeficiency virus type 1 genome into Gag virus-like particles in *Saccharomyces cerevisiae*

**DOI:** 10.1186/1475-2859-12-28

**Published:** 2013-03-26

**Authors:** Naoki Tomo, Toshiyuki Goto, Yuko Morikawa

**Affiliations:** 1 Kitasato Institute for Life Sciences and Graduate School for Infection Control, Kitasato University, Shirokane 5-9-1, Minato-ku, Tokyo, 108-8641, Japan; 2 School of Health Science, Faculty of Medicine, Kyoto University, Kawahara-cho 53, Shogoin, Sakyo-ku, Kyoto, 606-8507, Japan; 3 Present address: Life Technologies Japan Ltd, Mita Twin Bldg., Shibaura 4-2-8, Minato-ku, Tokyo, 108-0023, Japan

**Keywords:** Yeast, HIV, Virus-like particle, Genome packaging

## Abstract

**Background:**

Yeast is recognized as a generally safe microorganism and is utilized for the production of pharmaceutical products, including vaccines. We previously showed that expression of human immunodeficiency virus type 1 (HIV-1) Gag protein in *Saccharomyces cerevisiae* spheroplasts released Gag virus-like particles (VLPs) extracellularly, suggesting that the production system could be used in vaccine development. In this study, we further establish HIV-1 genome packaging into Gag VLPs in a yeast cell system.

**Results:**

The nearly full-length HIV-1 genome containing the entire 5^′^ long terminal repeat, U3-R-U5, did not transcribe gag mRNA in yeast. Co-expression of HIV-1 Tat, a transcription activator, did not support the transcription. When the HIV-1 promoter U3 was replaced with the promoter for the yeast glyceraldehyde-3-phosphate dehydrogenase gene, gag mRNA transcription was restored, but no Gag protein expression was observed. Co-expression of HIV-1 Rev, a factor that facilitates nuclear export of gag mRNA, did not support the protein synthesis. Progressive deletions of R-U5 and its downstream stem-loop-rich region (SL) to the gag start ATG codon restored Gag protein expression, suggesting that a highly structured noncoding RNA generated from the R-U5-SL region had an inhibitory effect on gag mRNA translation. When a plasmid containing the HIV-1 genome with the R-U5-SL region was coexpressed with an expression plasmid for Gag protein, the HIV-1 genomic RNA was transcribed and incorporated into Gag VLPs formed by Gag protein assembly, indicative of the *trans*-packaging of HIV-1 genomic RNA into Gag VLPs in a yeast cell system. The concentration of HIV-1 genomic RNA in Gag VLPs released from yeast was approximately 500-fold higher than that in yeast cytoplasm. The deletion of R-U5 to the *gag* gene resulted in the failure of HIV-1 RNA packaging into Gag VLPs, indicating that the packaging signal of HIV-1 genomic RNA present in the R-U5 to gag region functions similarly in yeast cells.

**Conclusions:**

Our data indicate that selective *trans*-packaging of HIV-1 genomic RNA into Gag VLPs occurs in a yeast cell system, analogous to a mammalian cell system, suggesting that yeast may provide an alternative packaging system for lentiviral RNA.

## Background

The yeast *Saccharomyces cerevisiae* has been used for many years as a model organism with which to study biological functions in higher eukaryotic cells. Such pioneering research has employed yeast genetics (e.g., gene-deletion mutant yeast) and molecular technologies (e.g., two-hybrid assay) and has uncovered fundamental cellular functions such as the cell cycle and mRNA turnover. Because of the accumulated knowledge of cell biology and systematic screening technologies, virologists have turned to the use of yeast as a model cell system to study the host factors required for the replication of higher eukaryotic viruses [1]. For example, bromo mosaic virus, a positive-strand RNA virus, has been shown to replicate and encapsidate its genome into virus particles in yeast [2], and the human papillomavirus genome has been shown to replicate stably in yeast [3]. The use of yeast genetic mutants has allowed to perform genome-wide screens to identify multiple host factors required for viral replication [4-6].

The applicability of yeast has been further expanded as cells for vaccine development, since yeast is recognized as generally safe and is utilized for the production of many pharmaceutical products. A good example is the hepatitis B surface antigen expressed in yeast, which is a safe and efficient vaccine used worldwide [7]. Another is the human papillomavirus capsid protein expressed in yeast, which is currently available as a vaccine [8,9]. Both viral proteins are self-assembled into virus-like particles (VLPs) in yeast expression systems, similar to mammalian and insect cell systems. Such VLPs are noninfectious but highly immunogenic because they mimic authentic viral particle structures. Consequently, VLPs represent new candidates for safe and efficacious vaccine components.

Human immunodeficiency virus type 1 (HIV-1), a member of the retrovirus family, is a causative agent for acquired immunodeficiency syndrome. The HIV-1 genomic RNA is reverse-transcribed into the cDNA and is integrated into the host cell chromosome. This cDNA form called proviral DNA is a template for transcription and replication of the HIV-1 genome. The proviral DNA has long terminal repeats (LTR) composed of unique region 3^′^ end (U3), repeat (R), and unique region 5^′^ end (U5) at both ends. These ends are important for viral transcription and replication: the U3 contains viral promoter and enhancer; the R contains a Tat-responsive region (TAR) and a poly A addition signal (pA); the U5 contains a primer binding site (PBS) [10]. The U3-R junction is the transcription start site. The 5^′^ LTR is followed by stem-loop (SL) structure-enriched untranslated region. SL1 and SL3 are a dimerization initiation signal (DIS) [11] and an encapsidation signal Psi for the HIV-1 genome [12,13], respectively, and both are absolutely required for HIV-1 genome packaging into viral particles. The three major genes, *gag*, *pol*, and *env*, encoding viral structural proteins, lie between the 5^′^ and 3^′^ LTRs. The *gag* gene encodes the viral capsid protein, Gag, which is essential for retroviral particle assembly. The *pol* and *env* genes encode viral specific enzymes and envelope proteins, both of which are necessary for multiple rounds of viral replication but are dispensable for viral particle production [14]. The HIV-1 genome also contains the accessory genes, *tat*, *rev*, *nef*, *vif*, *vpr*, and *vpu*, all of which contribute to efficient viral replication. The *tat* gene encodes Tat protein, which binds to the TAR sequence and is obligatory for HIV-1 transcription, whereas the *rev* gene encodes Rev protein, which binds to a highly structured RNA region, termed the Rev-responsive element (RRE), within the *env* gene and exports unspliced and incompletely spliced HIV-1 RNAs to the cytoplasm [15]. Thus, HIV-1 gene expression requires many RNA elements and the viral regulatory proteins Tat and Rev, whereas HIV-1 particle production requires only Gag.

Numerous protein expression systems, such as transfection with expression plasmids and infection with recombinant viral vectors, have shown that Gag protein expression alone in higher eukaryotic cells produces Gag VLPs, which are morphologically identical to the immature form of retroviral particles [16-19]. We previously showed that the expression of HIV-1 Gag protein in *S. cerevisiae* and the subsequent spheroplast formation produced Gag VLPs extracellularly [20]. We also showed that the Gag VLPs encased in yeast cell membrane induced innate immune responses (e.g., cytokine production), suggesting that the yeast production system has practical applications such as vaccine development [21]. Since the RNA elements required for HIV-1 genome packaging are well defined and distinct from the *gag* gene, it is possible that the addition of these RNA elements produces the HIV-1 VLPs containing the viral genome. In the present study, we tested this possibility and established *trans*-packaging of the HIV-1 genome into the Gag VLPs in a yeast cell system.

## Results

### Yeast did not support transcription or translation from HIV-1 LTR

**Figure 1:**
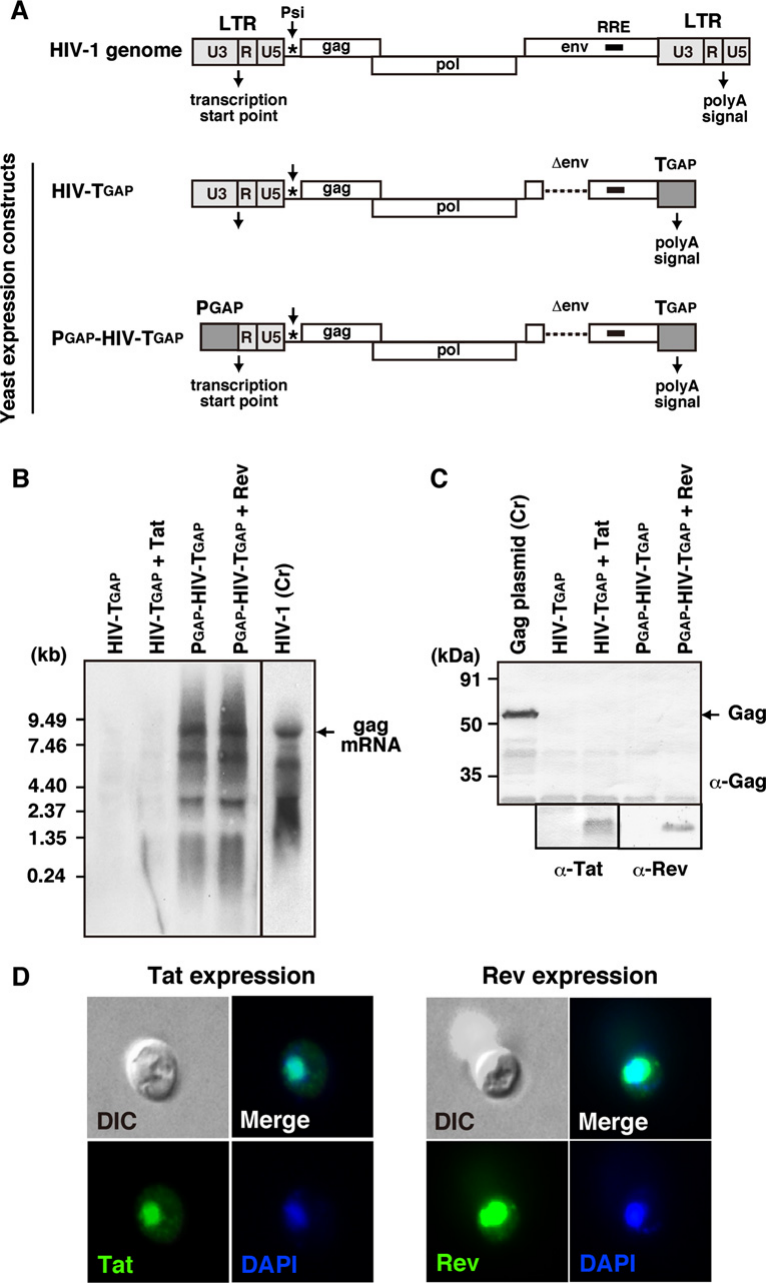
**No transcription or translation from HIV-1 LTR in yeast.** (**A**) Construction of yeast expression plasmids for HIV-1. In HIV-T_GAP_, the 3^′^ LTR of the HIV-1 cDNA (with deletion the *env* gene) was replaced with the terminator for the yeast GAP gene and was cloned into yeast 2μ plasmid pKT10. In P_GAP_-HIV-T_GAP_, the 5^′^ LTR U3 and 3^′^ LTR of HIV-1 cDNA (with deletion the *env* gene) were replaced by the promoter and terminator for the yeast GAP gene, respectively and were cloned into pKT10. (**B**) HIV-1 RNA synthesis in yeast transformed with HIV-1 expression plasmids. Yeast was transformed with HIV-T_GAP_, HIV-T_GAP_ plus HIV-1 Tat expression plasmids, P_GAP_-HIV-T_GAP_, or P_GAP_-HIV-T_GAP_ plus HIV-1 Rev expression plasmids. Northern blotting of the yeast transformants was performed using minus-strand biotinylated RNA probe for the HIV-1 *pol* gene. Representative blots were shown. HeLa cells transfected with HIV-1 molecular clone pNL43 were used as control. A 0.24-9.5 kb RNA ladder (GIBCO BRL) were used as molecular weight markers. (**C**) HIV-1 protein expression in yeast transformed HIV-1 expression plasmids. Yeast was transformed with HIV-1 expression plasmids as described in (**B**) and whole cell lysate was subjected to Western blotting using anti-HIV-1 p24 antibody. Yeast transformed with HIV-1 Gag expression plasmid was used as control. Representative blots were shown. Prestained 21–113 kDa protein markers (Bio-Rad) were used as molecular weight markers. (**D**) Expression of HIV-1 Tat and Rev in yeast. Yeast transformed with HIV-1 Tat and Rev expression plasmids were subjected to spheroplast formation and immunostaining using anti-HIV-1 Tat and Rev antibodies, respectively. Nuclei were stained with DAPI. Representative images were shown at the same magnification.

We initially analyzed the transcription of the HIV-1 LTR promoter in *S. cerevisiae*. The 5^′^ LTR of the HIV-1 cDNA (with deletion of the *env* gene for biosafety) was left intact, but the 3^′^ LTR was replaced with the terminator for the yeast glyceraldehyde-3-phosphate dehydrogenase (GAP) gene (Figure 1A). This chimeric DNA was cloned into yeast 2 μ plasmid pKT10 [22], and the yeast was transformed with the recombinant plasmid (referred to as HIV-T_GAP_). When RNA was purified from the transformants and subjected to Northern blotting using a minus-strand biotinylated RNA probe for the HIV-1 *pol* gene, little or no HIV-1 RNA was detected (Figure 1B). Co-transformation with a pRS plasmid containing the HIV-1 *tat* gene with an expression cassette (the promoter and terminator for yeast GAP) showed expression and nuclear localization of the HIV-1 Tat protein (Figure 1D), but the Tat protein did not transactivate the transcription of HIV-1 genome from the LTR (Figure 1B). These observations were consistent with previous reports showing that neither transcription from HIV-1 LTR nor transactivation by Tat occurred in *S. cerevisiae*[23,24].

We further replaced the U3 of the 5^′^ LTR (corresponding to the HIV-1 promoter) by the promoter for yeast GAP (referred to as P_GAP_-HIV-T_GAP_). Northern blotting revealed that this promoter rescued gag mRNA transcription (Figure 1B). The full-length gag mRNA was accompanied by some smaller RNA species, similar to the case with the transcription of proviral HIV-1 molecular clone pNL43 [25] observed in mammalian cells. Nonetheless, Gag protein expression was not observed when the whole cell lysates were analyzed by Western blotting using anti-HIV-1 p24 antibody, suggesting that HIV-1 expression was also blocked at post-transcriptional steps in yeast (Figure 1C). HIV-1 Rev binds to the RRE residing in HIV-1 RNA and facilitates the nuclear export of gag mRNA, unspliced mRNA containing RRE, in mammalian cells. It has been reported that Rev similarly functions dependently of RRE in yeast [26,27]. However, the co-expression of Rev from a pRS plasmid containing the *rev* gene with an expression cassette (the promoter and terminator for yeast GAP) (Figure 1D) did not support Gag protein expression (Figure 1C), suggesting, although not proving, that HIV-1 gag mRNA containing R-U5 was not efficiently translated in yeast.

### The R-U5-SL region to the gag start ATG codon inhibited Gag protein expression in yeast

**Figure 2:**
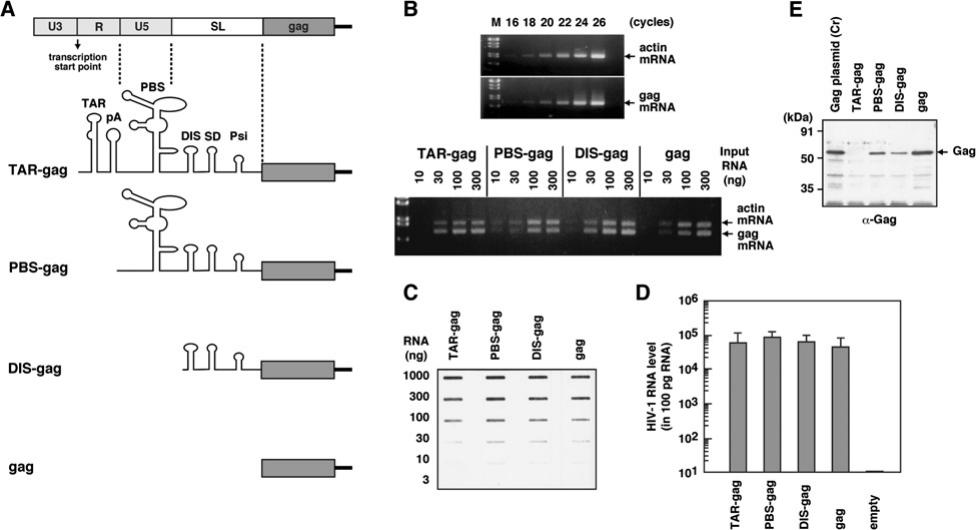
**Inhibition of HIV-1 Gag protein expression by the R-U5 and SL regions in yeast.** (**A**) Schematical representation of HIV-1 expression plasmids with deletions in the R-U5 and SL regions. (**B**) Semi-quantitative RT-PCR for HIV-1 RNA in yeast. Total cellular RNA was isolated from yeast transformed with HIV-1 expression plasmids with deletions in the R-U5 and SL and was subjected to semi-quantitative RT-PCR for HIV-1 gag mRNA and yeast actin mRNA. Using 300 ng of total cellular RNA, RT-PCR for HIV-1 gag mRNA and yeast actin mRNA was performed up to 26 PCR cycles (upper). Using a series of dilutions of the cellular RNA (10 to 300 ng), RT-PCR for HIV-1 gag mRNA and yeast actin mRNA was performed at 20 PCR cycles (lower). Representative blots were shown. The PCR product for HIV-1 gag mRNA corresponds to 670 bases and that for yeast actin mRNA 840 bases. (**C**) Semi-quantification of HIV-1 RNA in yeast. Total cellular RNA of the yeast transformants was serially diluted (3 to 1000 ng), slot-blotted, and detected by hybridization with the minus-strand RNA probe for the HIV-1 *pol* gene. Representative blots were shown. (**D**) Real-time RT-PCR for HIV-1 RNA in yeast. Total cellular RNA isolated from yeast transformants was reverse-transcribed and aliquots of the reaction samples were used for amplification up to 40 PCR cycles with specific primers for HIV-1 gag mRNA. Empty indicates total cellular RNA isolated form yeast transformant with an empty pKT10 plasmid (negative control). Data were shown as means with standard deviations from 3 independent experiments. (**E**) HIV-1 Gag protein expression in yeast. Whole cell lysates of yeast transformed with HIV-1 expression plasmids with deletions in the R-U5 and SL regions were subjected to Western blotting using and anti-HIV-1 p24 antibody. Representative blots were shown.

HIV-1 gag mRNA is identical to its genomic RNA and contains the R-U5-SL region at its 5^′^ end. It is well documented that the R-U5-SL region forms a highly folded and complicated structure [28-31]. To define the regions that inhibited the translation of HIV-1 gag mRNA in yeast, a series of 5^′^ truncations was carried out in the R-U5-SL region (Figure 2A), and the constructs were similarly cloned into a pKT10 plasmid with a yeast expression cassette (the promoter and terminator for yeast GAP) [22]. When the whole cell lysates were subjected to Western blotting using anti-HIV-1 p24 antibody, the construct containing the full length of the R-U5-SL (referred to as TAR-gag) showed no Gag protein expression. In contrast, 5^′^ truncations in the R-U5-SL (referred to as PBS-gag and DIS-gag) partially restored Gag protein expression. The Gag protein level produced by the DIS-gag was lower than that by the PBS-gag, in accordance with previous reports on strong inhibition of gag mRNA translation by the TAR [32-34] and SL regions [35]. Complete deletion of the R-U5-SL (referred to as gag) fully restored Gag protein expression when compared with yeast transformed with the Gag expression plasmid containing the HIV-1 *gag* gene alone (Figure 2E).

Total cellular RNA was isolated from these yeast transformants and was subjected to semi-quantitative RT-PCR analysis for HIV-1 gag mRNA and yeast actin mRNA. When the amplification kinetics were initially monitored with 300 ng of cellular RNA, we found that the RT-PCR products for both HIV-1 gag mRNA and yeast actin mRNA increased as the number of PCR cycles increased (up to 26 cycles) (Figure 2B, upper). Using a series of dilutions of the cellular RNA, the product yields at 20 PCR cycles indicated that all 5^′^ truncation constructs produce largely similar levels of HIV-1 gag mRNA (Figure 2B, lower). For the direct detection of RNA, a series of dilutions of the cellular RNA was slot-blotted on the membrane, followed by hybridization with the RNA probe for the HIV-1 *pol* gene. The results confirmed that the 5^′^ truncation constructs produced largely equivalent levels of HIV-1 gag mRNA (Figure 2C). Real-time RT-PCR analysis further confirmed these findings (Figure 2D). These data indicate that the HIV-1 R-U5-SL region that generates a highly structured RNA has an inhibitory effect on Gag translation in yeast, as reported in mammalian cells [35,36].

### *Trans*-packaging of HIV-1 genomic RNA into Gag VLPs in a yeast cell system

In mammalian cells, HIV-1 gag mRNA is also used as viral genomic RNA and is incorporated into viral particles. The genomic RNA/gag mRNA has the encapsidation signal Psi in the SL region, which is absolutely required for viral genome packaging into HIV-1 particles [37-39]. The SL region also includes a signal, termed dimerization initiation site (DIS), which is essential for the dimerization of HIV-1 genomic RNA and overlaps with a signal required for the packaging of the genomic RNA, suggesting that the dimerization and packaging processes are possibly coupled [40,41]. Several studies have indicated that other RNA elements (e.g., TAR and PBS) are also involved in the efficiency of HIV-1 genome packaging [38,42,43]. From these studies, we considered that the use of the same construct for the synthesis of HIV-1 genomic RNA and Gag protein (i.e., *cis*-packaging) was a less effective method for the RNA packaging into Gag VLPs in the yeast cell system.

**Figure 3:**
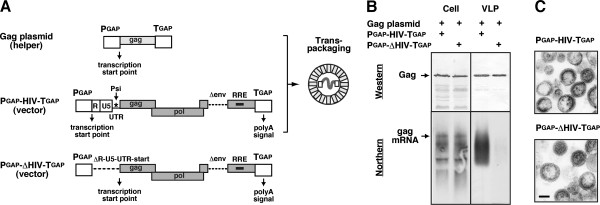
***Trans*-packaging of HIV-1 genomic RNA into Gag VLPs in yeast.** (**A**) Schematical representation of HIV-1 expression plasmids used for *trans*-packaging. HIV-1 Gag expression plasmid was used for production of Gag VLPs (as a helper plasmid). P_GAP_-HIV-T_GAP_ and P_GAP_-ΔHIV-T_GAP_ were used for synthesis of HIV-1 genomic RNA (as vector plasmids). (**B**) Production of HIV-1 Gag VLPs and packaging of HIV-1 RNA in yeast. Yeast was cotransformed with the helper and vector plasmids. After removal of the cell wall, yeast spheroplasts were cultured overnight for Gag VLP release. Cells and purified Gag VLPs were analyzed by Western blotting using anti-HIV-1 p24 antibody. RNA was isolated from cells and purified Gag VLPs and was analyzed by Northern blotting using minus-strand biotinylated RNA probe for the HIV-1 *pol* gene. All blots are representative from 3–4 independent experiments. (**C**) Electron microscopy of Gag VLPs. Purified Gag VLPs were analyzed by electron microscopy. Representative micrographs were shown at the same magnification. Bar =100 nm.

It is well known that the synthesis of retroviral genomic RNA can be separated from that of Gag protein, and that retroviral genomic RNA transcribed from a construct is incorporated into Gag VLPs produced by another construct in mammalian cells (i.e., *trans*-packaging) [44,45]. This method is extensively used for the production of viral gene therapy vectors [46,47]. We investigated whether or not *trans*-packaging is possible in a yeast cell system. To this end, a plasmid containing the HIV-1 genome with the R-U5-SL region (as a vector plasmid) was co-expressed with the Gag expression plasmid containing the *gag* gene alone (as a helper plasmid) in yeast. For comparison, a plasmid containing the HIV-1 genome without the R-U5-SL region or the gag start codon was similarly coexpressed with the Gag expression plasmid (Figure 3A). Following removal of the cell wall, yeast spheroplasts were maintained under an isotonic condition overnight, and Gag VLPs were purified from the culture medium by ultracentrifugation, as described previously [20,48]. Western blotting of the VLP fractions with anti-HIV-1 p24 antibody confirmed the production of Gag VLPs. When RNA was isolated from equivalent volumes of the Gag VLP fractions and was subjected to Northern blotting with the *pol* RNA probe, we found the presence of HIV-1 genomic RNA in the VLP fractions in coexpression with the HIV-1 genome with the R-U5-SL region. In contrast, no substantial level of HIV-1 genomic RNA was observed in the VLPs obtained in coexpression with the HIV-1 genome lacking the R-U5-SL region, although the level of HIV-1 RNA in the cells was comparable (Figure 3B). It is known that the retrovirus genomic RNA in viral particles is randomly nicked and shows smear bands under denatured conditions although it is intact in cells [49,50].

Electron microscopy confirmed no morphological differences in these Gag VLPs (Figure 3C). These data indicate that *trans*-packaging of HIV-1 genomic RNA into Gag VLPs is possible in a yeast cell system and is dependent on the presence of the R-U5-SL region, similar to the case with mammalian cell systems.

### Selective packaging of HIV-1 genomic RNA into Gag VLPs in a yeast cell system

**Figure 4:**
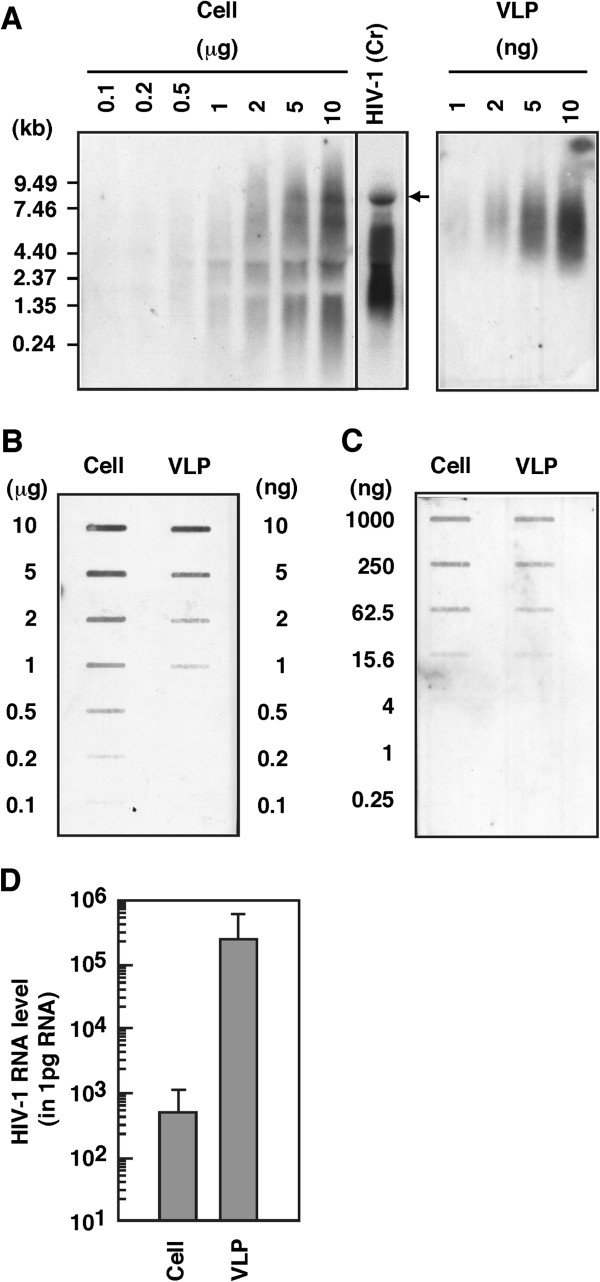
**Preferential incorporation of HIV-1 genomic RNA into Gag VLPs in yeast.** (**A**) Efficiency of incorporation of HIV-1 genomic RNA into Gag VLPs in yeast. Yeast was cotransformed with the Gag expression plasmid (helper) and P_GAP_-HIV-T_GAP_ plasmid (vector). After spheroplast formation, cells were cultured overnight for Gag VLP release from yeast spheroplasts were carried out as before. RNA was isolated from yeast spheroplasts and Gag VLPs, serially diluted, and analyzed by Northern blotting using minus-strand biotinylated RNA probe for the HIV-1 *pol* gene. Representative blots were shown. HeLa cells transfected with HIV-1 molecular clone pNL43 were used as control. Arrow indicates gag mRNA. A 0.24-9.5 kb RNA ladder (GIBCO BRL) were used as molecular weight markers. (**B**) Semi-quantification of incorporation efficiency of HIV-1 genomic RNA into Gag VLPs. RNAs of the yeast spheroplasts and Gag VLPs were serially diluted, slot-blotted, and detected by with hybridization with the minus-strand RNA probe for the HIV-1 *pol* gene. Representative blots were shown. (**C**) Semi-quantification of incorporation efficiency of yeast actin mRNA into Gag VLPs. RNAs of the yeast spheroplasts and Gag VLPs were serially diluted, slot-blotted, and detected by with hybridization with the minus-strand RNA probe for yeast actin mRNA. Representative blots were shown. (**D**) Quantification of incorporation efficiency of HIV-1 genomic RNA into Gag VLPs by real-time RT-PCR. RNA was isolated from yeast spheroplasts or Gag VLP and was reverse-transcribed. Aliquots of the reaction samples were used for amplification up to 40 PCR cycles with specific primers for unspliced HIV-1 mRNA. Data were shown as means with standard deviations from 3 independent experiments.

We investigated to what extent HIV-1 genomic RNA was selectively packaged into Gag VLPs. The total cellular RNA and the VLP RNA were serially diluted and analyzed by Northern blotting using the *pol* RNA probe (Figure 4A). The RNA dilutions (from 10 to 0.1 μg in the case of total cellular RNA; from 10 to 0.1 ng in the case of VLP RNA) were also subjected to slot blotting and probed with the RNA probe. When the endpoint dilutions were compared, they were at 0.2 μg for the cellular RNA but at 1 ng for the VLP RNA, indicating that the HIV-1 genomic RNA in Gag VLPs was concentrated by approximately 500-fold compared to the same RNA in yeast cells (Figure 4B). For more accurate analysis, the unspliced HIV-1 RNA (containing the SL region) present in the cellular RNA and VLP RNA fractions was quantified by real-time RT-PCR. The results revealed similarly approximately 500-fold concentration of HIV-1 genomic RNA in the VLP fraction (Figure 4D). In contrast, when the RNA dilutions were slot-blotted and probed with the minus-strand RNA probe for yeast actin mRNA, no preferential incorporation into Gag VLPs was observed (Figure 4C). This is consistent with a previous study, in which the majority of cellular RNAs were nonselectively incorporated into retrovirus particles [51]. Together, these data indicate selective packaging of HIV-1 genomic RNA into Gag VLP in a yeast cell system.

### Transgene expression by Gag VLPs in mammalian cells

**Figure 5:**
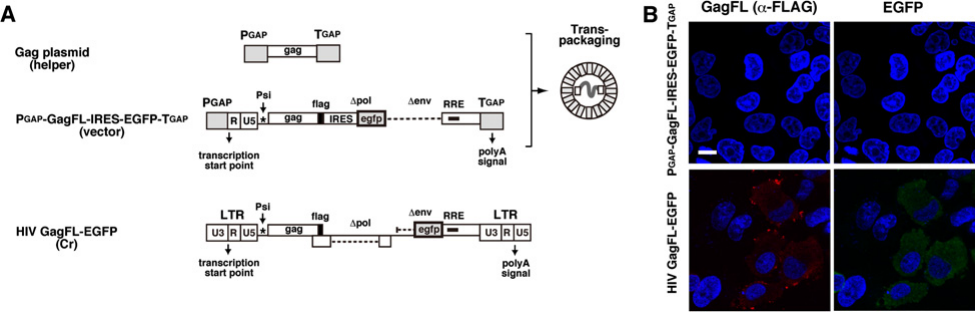
**Defects in gene expression of Gag VLPs containing a bicistronic expression cassette in target cells.** (**A**) Schematical representation of expression plasmids used for *trans*-packaging and subsequent protein expression. HIV-1 Gag expression plasmid and P_GAP_-GagFL-IRES-EGFP-T_GAP_ were used for production of Gag VLPs (as a helper plasmid) and synthesis of reporter RNA (as a vector plasmid), respectively. HIV GagFL-EGFP was used as a control for GagFL and EGFP expression in mammalian cells. (**B**) Expression of HIV-1 GagFL and EGFP in HeLa cells. Yeast was cotransformed with the helper and vector plasmids. After removal of the cell wall, yeast spheroplasts were cultured overnight for Gag VLP release as before. Produced Gag VLPs (equivalent to 1 μg RNA) were added to HeLa cells. Following incubation at 37°C for 2 days, HeLa cells were subjected to immunostaining using anti-FLAG (for detection of GagFL) antibody. Nuclei were stained with DAPI. Representative images were shown at the same magnification. Bar, 10 μm.

We finally tested whether the HIV-1 genomic RNA packaged into yeast-produced Gag VLPs was expressed in mammalian cells. The bicistronic reporter construct that composed of the *gag* gene fused with a FLAG epitope tag sequence, the IRES sequence derived from encephalomyocarditis virus, and the gene for enhanced green fluorescent protein (*egfp*) was generated in a pNL43 derivative with deletions of the *pol* gene and the *env* gene. In this construct, GagFL is translated in a cap-dependent fashion, whereas EGFP is in a cap-independent fashion under control of the IRES. The 3^′^ LTR and the U3 of the 5^′^ LTR were replaced by the terminator and the promoter for the yeast GAP gene, respectively, and the resultant construct was cloned into yeast 2μ plasmid pKT10 [22] (referred to as P_GAP_-GagFL-IRES-EGFP-T_GAP_). For *trans*-packaging of this bicistronic reporter RNA into Gag VLPs, P_GAP_-GagFL-IRES-EGFP-T_GAP_ (as a vector plasmid) was co-expressed with the Gag expression plasmid containing the *gag* gene (as a helper plasmid) in yeast (Figure 5A). Yeast spheroplast formation and Gag VLP production were carried out as before. HeLa cells were incubated with the Gag VLPs for 2 days and were subjected to immunostaining with anti-FLAG antibody. Neither GagFL nor EGFP was detected by confocal microscopy (Figure 5B, upper). We also used Raw264.7 cells (mouse monocytic macrophage-like cell line) because macrophage-like cells endocytose exogenously added antigens more efficiently than epithelial cells, but we failed to see expression of GagFL and EGFP (data not shown). In contrast, transfection with the pNL43 derivative cDNA containing the *gag* gene fused with a FLAG sequence and the *egfp* gene exhibited expression of GagFL and EGFP (Figure 5B, lower).

## Discussion

### Transcription of HIV-1 genome in yeast

The 5^′^ LTR of HIV-1 is composed of the U3-R-U5 and is followed by the SL region. The U3 region is a promoter and contains the binding sites for transcription factors, such as nuclear factor κB, Sp1, and AP2. However, HIV-1 transcription is regulated primarily by Tat protein. Tat binds to the TAR sequence within the R region of HIV-1 mRNA transcripts and recruits the host positive transcription elongation factor b (P-TEFb) complex containing cyclin-dependent kinase 9 and cyclin T1. The binding of the complex facilitates transcription of the provirus by cellular RNA polymerase II. In the absence of Tat, only short transcripts are generated because RNA polymerase II is readily dissociated from the DNA template [15]. In this study, we found in *S. cerevisiae* that Tat protein overexpression did not support transcription from the LTR but that replacement of the U3 region by the promoter for yeast GAP did support the transcription (Figure 1), likely because the yeast promoter is Tat-independent as opposed to the HIV-1 promoter. It has also been suggested that cyclin T1 is a species-specific cofactor for HIV-1 transcription, since mouse cyclin T1 does not support HIV-1 transcription due to a single amino acid change [52,53].

### Translation of HIV-1 mRNA in yeast

The HIV-1 gag mRNA contains the R-U5 and SL regions upstream from the gag start ATG codon. This 5^′^ untranslated region (332 bases) has been suggested to form a highly folded RNA structure [29-31]. In cell-free systems and *Xenopus* oocytes, the 5^′^ untranslated region, especially the TAR region, inhibits cap-dependent translation of the *gag* gene [32-34,36]. A recent study showed that in 293 cells the SL region, especially the Psi site, was the major determinant of translation inhibition [35]. Consistent with these studies, we found that in yeast, (i) the 5^′^ untranslated region had an inhibitory effect on the Gag translation without reducing in the gag mRNA levels and (ii) no translation was observed when the untranslated region included the TAR region (Figure 2).

The 5^′^ untranslated region is required for packaging of HIV-1 genome into viral particles. Nevertheless, the 5^′^ untranslated region, if tested experimentally, inhibits its downstream Gag translation, which is required for virus particle production. To explain this discrepancy, some hypotheses have been proposed. One is the alternating structure model of the 5^′^ HIV-1 RNA, termed long-distance interaction (LDI) and branched multiple hairpin (BMH): the equilibrium between the two was hypothesized to regulate RNA dimerization, packaging, and translation [28,54,55]. However, mutations to alter the LDI-BMH equilibrium did not affect translation efficiency [56]. Another possibility is internal ribosome entry site (IRES) activity within the 5^′^ untranslated region of the HIV-1 RNA. An earlier study did not identify putative IRES [36] and later studies found IRES activity in a lentiviral family [57,58]. Interestingly, HIV-1 IRES functions only at the G2/M transition phase of the cell cycle [57], although IRES-dependent Gag translation is still controversial. In *S. cerevisiae*, IRES-mediated translation has been observed with endogenous yeast genes as well as the IRES elements of hepatitis C virus [59], but neither poliovirus nor encephalomyocarditis virus IRES can function [60,61]. In the present study, deletion of the entire 5^′^ untranslated region (R-U5-SL) fully restored Gag translation. Thus, our study argues against the IRES-mediated Gag translation although we cannot rule out a failure of the IRES activity in yeast. It should be noted that in HIV-1 infected cells, the gag mRNA is efficiently translated to Gag protein even though it contains the 5^′^ untranslated region. It is possible that a host factor(s), for examples, to unwind the 5^′^ RNA structures is involved in gag mRNA translation in infected cells. We suggest that complementation of host factors, especially infection-induced host factors, to this yeast cell system would identify such factors for gag mRNA translation.

### Genome packaging into Gag VLPs in yeast and the benefits of yeast-derived Gag VLPs encapsidating genes

Results from numerous mutagenesis studies indicate that each RNA element within the 5^′^ untranslated region is responsible for packaging of HIV-1 genome. For example, mutations at the SL region (the DIS and Psi sites and the gag start codon) severely impaired HIV-1 genome packaging [38,39] and mutations at the TAR, pA, and PBS regions similarly impaired the genome packaging [40,42], indicating that the entire 5^′^ untranslated region is involved in efficient packaging of HIV-1 genome. These studies also suggest that the structures, but not the specific sequences, of the 5^′^ untranslated region are important for packaging of HIV-1 genome. In our study, the TAR-gag construct that contained the full length of the R-U5-SL showed no Gag protein expression. When the 5^′^ untranslated region was progressively truncated, the Gag protein was expressed but the levels varied in the truncations. From these results, we suppose that the *cis*-packaging efficiencies of these 5^′^ truncated RNAs are difficult to evaluate in a yeast cell system.

In contrast, our study indicated that HIV-1 genomic RNA was preferentially incorporated into Gag VLPs in yeast when Gag was supplied *in trans*. Generally, the technology of *trans*-packaging of gene is used to produce replication-deficient viral vectors, which are much safer than replication-competent viral vectors produced by *cis*-packaging [44,45]. In the field of vaccinology, various types of viral vaccines have been developed. Live attenuated viruses and recombinant viral vectors are the most effective vaccines and stimulate cellular as well as humoral immune responses. They replicate and express their antigens in cells, but safety concerns cannot be excluded. In contrast, viral protein components and peptides are safe but often lack the ability to induce cellular immunity. VLPs are highly assembled structures of viral protein components, mimicking the authentic virion without including viral genome. Thus, they are not infectious but often effective at stimulating cellular immunity [62]. In fact, our previous study showed that the Gag VLPs encased in yeast cell membrane induced maturation and cross-presentation of dendritic cells [21]. However, because VLPs do not contain genetic materials, they do not endogenously produce intracellular antigens that stimulates cellular immunity by the major histocompatibility complex I antigen presentation pathway. Also, they usually do not contain viral envelope glycoproteins that are major immunogens to elicit neutralizing antibodies. Since yeast does not trim the glycans to produce hyper-mannosylated glycans, it may not be readily available for production of VLPs presenting glycoproteins on the VLP membrane at present [63]. Our data suggest the possibility that noninfectious VLPs encapsidating gene of interest can be produced in a yeast cell system and may provide clues to the development of yeast VLP vaccines that confer ensured safety and enhanced immunogenicity. For this purpose, we produced Gag VLPs packaging the bicistronic reporter gene cassettes (i.e., *gagfl* and *egfp*) in a yeast cell system and tested expression of the reporter genes transduced by Gag VLPs in HeLa and Raw264.7 cells. In this bicistronic construct, GagFL is translated in a cap-dependent fashion, whereas EGFP is in a cap-independent fashion. However, we did not see expression of GagFL or EGFP (Figure 5B, upper). We finally isolated the RNA from the Gag VLPs and transfected the RNA to HeLa cells but failed to see GagFL and EGFP expression (data not shown). These results suggested that the failure of the transgene expression was not due to target cell types or the uncoating ability of Gag VLPs. Rather, it is ascribable to extensive nicking of the RNA within Gag VLPs. Retroviral/lentiviral gene expression requires reverse transcription of viral RNA and subsequent integration of the cDNA into host cell chromosomes before its transcription and translation. The input viral RNA is not directly used as mRNA and viral protein expression occurs only from the integrated proviral cDNA. The reason is not known but it may be partly that the RNA in viral particles is randomly nicked [49,50]. However, we believe that mRNA transduction without integration would be safer and more suitable for vaccine design than stable integration of transgene into chromosomes. Further studies are needed to develop the methods to incorporate intact mRNA into Gag VLPs (e.g., by using non-cognate packaging signals, by shortening transgene RNA, or by forming stable RNA-protein complex like mature Gag capsid).

## Conclusions

Cells of the yeast *Saccharomyces cerevisiae* have been used to develop VLP vaccines (e.g., hepatitis B virus and human papillomavirus). Such VLPs are considered new candidates for safe and efficacious vaccine components because they are noninfectious and highly immunogenic. Yeast cell systems have also been used as model cell systems with which to study host factors required for the replication of higher eukaryotic viruses. Bromo mosaic virus, a positive-strand RNA virus, and human papillomavirus, a DNA virus, have been shown to replicate and encapsidate their genomes into virus particles in yeast. We previously demonstrated the production of Gag VLPs in yeast spheroplasts. Our present study established the *trans*-packaging of the HIV-1 genome into Gag VLPs in a yeast cell system. This study also revealed that the 5^′^ untranslated region of the HIV-1 genome (the R-U5-SL region) inhibited its downstream translation (Gag protein expression). This yeast system may be useful for the study of HIV-1 genome packaging and translation.

## Methods

### Plasmid construction and yeast expression

A full-length HIV-1 cDNA molecular clone, pNL43 [25], was used for DNA construction. A Kpn I-Nhe I fragment of the *env* gene (nucleotide positions 6343–7250) of pNL43 was initially deleted (for biosafety), and the 3^′^ LTR was replaced by the terminator for the yeast GAP gene. The chimeric HIV-1 DNA was cloned into yeast 2 μ plasmids containing the *URA3* gene as a selective marker (referred to as HIV-T_GAP_). The U3 of the 5^′^ LTR (HIV-1 promoter) was further replaced by the constitutive promoter for the yeast GAP gene (referred to as P_GAP_-HIV-T_GAP_). The entire 5^′^ LTR and its downstream SL region, including the gag start codon, were also deleted in the context of P_GAP_-HIV-T_GAP_ (referred to as P_GAP_-ΔHIV-T_GAP_). A series of truncations of the 5^′^ LTR and its downstream SL regions was carried out by PCR using relevant forward and reverse primers. For the expression of Gag protein *in trans*, the full-length *gag* gene was placed under the control of the GAP promoter, followed by the GAP terminator, and the expression cassette was cloned into yeast 2 μ plasmid pRS423 containing the *HIS3* gene as a selective marker. For the expression of Tat and Rev proteins, the exons of the tat and rev genes were joined by overlapping PCR. The PCR fragments were similarly placed between the GAP promoter and the GAP terminator and were cloned into yeast 2 μ plasmid pRS423/424 containing the *HIS3/TRP1* gene as a selective marker. *S. cerevisiae* strain RAY3A-D (*MATa/*α *ura3/ura3 his3/his3 leu2/leu2 trp1/trp1*) [64] was transformed by the recombinant plasmids. The transformants were inoculated in the appropriate synthetic medim and grown at 30°C.

For protein expression in mammalian cells, the bicistronic IRES construct (the *gag* gene fused with a FLAG epitope tag sequence, the IRES sequence derived from encephalomyocarditis virus [Clontech], and the gene for *egfp*) was initially generated in the pNL43 derivative with deletions of the *pol* gene (nucleotide positions 2290–4553) and the Kpn I-Nhe I fragment of the *env* gene (nucleotide positions 6343–7250). The 3^′^ LTR and the U3 of the 5^′^ LTR were replaced by the terminator and the promoter for the yeast GAP gene, respectively, and the resultant construct was cloned into yeast 2 μ plasmid pKT10 [22] (referred to as P_GAP_-GagFL-IRES-EGFP-T_GAP_). The pNL43 derivative expressing the Gag protein fused with a FLAG epitope tag and EGFP was similarly generated from the pNL43 derivative with deletions of the *pol* and *env* genes.

### Preparation of yeast spheroplasts and production of Gag VLPs

The procedure for yeast spheroplast formation was described previously [20]. Yeast transformants were grown at 30°C in synthetic defined medium (0.67% yeast nitrogen base, 2% glucose, and amino acid mixtures) without uracil, histidine, and/or tryptophane. Yeast cells were suspended in wash buffer (50 mM Tris [pH 7.5], 5 mM MgCl_2_, and 1 M sorbitol) containing 30 mM DTT and incubated at 30°C for 20 min with gentle shaking. The cells were resuspended in wash buffer containing 3 mM DTT and 0.4 mg/ml Zymolyase and were incubated at 30°C for 20 min for cell wall digestion. After being washed twice with 1 M sorbitol, spheroplasts were cultured in YPD (1% yeast extract, 2% peptone, and 2% glucose) medium containing 1 M sorbitol at 30°C overnight with gentle shaking (at 60 rpm).

Yeast-produced Gag VLPs were purified as described previously [20]. Briefly, the culture medim of yeast spheroplasts was clarified and centrifuged through 30% (w/v) sucrose cushions in an SW28 rotor (Beckman Coulter) at 120,000 × g for 1.5 hr at 4°C. The VLP pellets were resuspended in phosphate-buffered saline and were centrifuged on 20-70% (w/v) sucrose gradients in an SW55 rotor (Beckman Coulter) at 120,000 × g overnight at 4°C.

### Expression in mammalian cells

HeLa and Raw264.7 cells were grown in Eagle’s minimum essential medium supplemented with 10% fetal bovine serum. Gag VLPs (equivalent to 1 μg RNA) were added to HeLa and Raw264.7 cells. Transfection with RNA isolated from Gag VLPs or plasmid DNA was carried out using Lipofectamine 2000 (Invitrogen).

### Western blotting

Yeast cells (0.5 OD) were separated by SDS-PAGE. Western blotting was carried out using anti-HIV-1 p24CA, anti-HIV-1 Tat, and anti-HIV-1 Rev mouse monoclonal antibodies (Advanced Biotechnologies).

### Semi-quantitative and quantitative RT-PCRs

Total cellular RNA and VLP RNA were isolated with the RNeasy kit (Qiagen) according to the manufacturer’s instructions. Contaminant DNA was digested with DNase I during the isolation. Semi-quantitative RT-PCR was performed with the ReverTra Dash kit (Toyobo) according to the manufacturer’s instructions. For cDNA synthesis, RNA was mixed with random primers and the reaction was carried out at 42°C for 20 min. For amplification of the cDNA, aliquots of the RT reaction samples were mixed with 100 nM of each primer, and a three-step reaction (98°C for 10 sec, 60°C for 2 sec, and 74°C for 30 sec) was cycled. The following primer sets were used: 5^′^-ATGGGTGCGAGAGCGTCGGTATTAAGC-3^′^ and 5^′^-CAATAGGCCCTGCATGCACTGGATG-3^′^ for HIV-1 *gag* and 5^′^-GCCCCAGAAGAACACCCTGTTCTTT-3^′^ and 5^′^-TTAGAAACACTTGTGGTGAACGATA-3^′^ for yeast actin mRNAs.

Real-time RT-PCR was performed with PrimeScript RT reagent kit (Takara) and subsequently with SYBR Green Realtime PCR Master Mix (Toyobo). For cDNA synthesis, 1 μg of RNA was mixed with a mixture of oligo dT and random primers (supplied by the RT kit) and the reaction was carried out at 37°C for 15 min according to the manufacturer’s instruction. For amplification of the cDNA, 1/100^th^ of aliquots of the reaction samples were mixed with 100 nM of each primer and two-step reaction (95°C for 5 sec and 60°C for 30 sec) was cycled. The following primer sets were used and produced single amplification products (confirmed by melting curve analysis): 5^′^-GCTTGCTGAAGCGCGCACGG-3^′^ and 5^′^-GACGCTCTCGCACCCATCTC-3^′^ for unspliced HIV-1 (nucleotide positions 701–806) [65] and 5^′^-ATAATCCACCTATCCCAGTAGGAGAAAT-3^′^ and 5^′^-TTTGGTCCTTGTCTTATGTCCAGAATGC-3^′^ for HIV-1 *gag* (nucleotide positions 1544–1658) [66] mRNAs. Relative quantification of HIV-1 RNA was performed in reference to a standard curve prepared by amplification of 10-fold serial dilutions (50–0.05 pg) of pNL43 [25].

### Northern blotting and slot blotting

Minus-strand RNA probes were synthesized with Maxi script T7 kit (Ambion) according to the manufacturer′s instructions. The fragments of HIV-1 *pol* and yeast actin genes (nucleotide positions 3826–4160 and 579–1436, respectively) were cloned into pGEM3 vector (Promega) and were *in vitro*-transcribed with biotinylated UTP (Roche) at 37°C for 60 min. After digestion of the DNA templates with DNase I, RNA transcripts were purified using a Quick Spin Column (Roche).

For Northern blotting, RNA samples were denatured, electrophoresed in 0.8% agarose gels, and blotted onto Hybond N^+^ membrane (Amersham). Hybridization and detection were carried out with the Ultrahyb kit (Ambion) and the Biotin Luminescent Detection kit (Roche) according to the manufacturer’s instructions, respectively. Briefly, hybridization with RNA probes was performed at 68°C overnight and washing was performed first with 2×SSC buffer containing 0.1% SDS and then with 0.1×SSC buffer at 68°C. For slot-blot analysis, a series of dilutions of RNA samples was blotted onto Hybond N^+^ membrane by vacuuming and hybridization and detection were similarly carried out.

### Immunofluorescent staining

Yeast cells were fixed in 3.7% formalin in YPD at 30°C for 30 min. Following removal of the cell wall, spheroplasts were treated with 70% ethanol at 4°C for 5 min for membrane permeabilization. After blocking with 0.1% BSA, cells were incubated with anti-HIV-1 Tat or Rev mouse monoclonal antibodies and subsequently with anti-mouse IgG-Alexa Fluor 488 (Molecular Probes). Nuclei were stained with 4^′^, 6-diamidino-2-phenylindole dihydrocloride (DAPI).

HeLa nad Raw264.7 cells were fixed in 3.7% formalin in phosphate-buffered saline for 30 min and treated with 0.1% Triton-X 100 for 10 min for membrane permeabilization. After blocking with 0.1% BSA, cells were incubated with anti-FLAG mouse monoclonal antibody (Sigma) and subsequently with anti-mouse IgG-Alexa Fluor 568 (Molecular Probes). Nuclei were stained with DAPI. Cells were observed with a laser-scanning confocal microscope (TCS-SP5, Leica).

### Electron microscopy

Purified Gag VLP pellets were fixed in 2% glutaraldehyde in 50 mM cacodylate buffer (pH 7.2) for 2 hr and postfixed with 1% osmium tetroxide for 1 hr. The pellets were embedded in epoxy resin. Ultrathin sections were stained with uranyl acetate and lead citrate and were examined with an electron microscope.

## Abbreviations

DIS: Dimerization initiation signal; GAP: Glyceraldehyde-3-phosphate dehydrogenase; HIV-1: Human immunodeficiency virus type 1; LTR: Long terminal repeats; pA: Poly A addition signal; PBS: Primer-binding site; RRE: Rev-responsive element; SL: Stem-loop; TAR: Tat-responsive element; VLP: Virus-like particle

## Competing interests

The authors declare that they have no competing interests.

## Authors’ contributions

NT carried out the biochemical and microscopy studies including construction of plamids. TG carried the electron microscopy. YM designed the experiments and wrote the manuscript. All authors read and approved the manuscript.
